# Mutations of Different Molecular Origins Exhibit Contrasting Patterns of Regional Substitution Rate Variation

**DOI:** 10.1371/journal.pcbi.1000015

**Published:** 2008-02-29

**Authors:** Navin Elango, Seong-Ho Kim, NISC Comparative Sequencing Program, Eric Vigoda, Soojin V. Yi

**Affiliations:** 1 School of Biology, Georgia Institute of Technology, Atlanta, Georgia, United States of America; 2 Genome Technology Branch and NIH Intramural Sequencing Center, National Human Genome Research Institute, National Institutes of Health, Bethesda, Maryland, United States of America; 3 College of Computing, Georgia Institute of Technology, Atlanta, Georgia, United States of America; Stanford University, United States of America

## Abstract

Transitions at CpG dinucleotides, referred to as “CpG substitutions”, are a major mutational input into vertebrate genomes and a leading cause of human genetic disease. The prevalence of CpG substitutions is due to their mutational origin, which is dependent on DNA methylation. In comparison, other single nucleotide substitutions (for example those occurring at GpC dinucleotides) mainly arise from errors during DNA replication. Here we analyzed high quality BAC-based data from human, chimpanzee, and baboon to investigate regional variation of CpG substitution rates.

We show that CpG substitutions occur approximately 15 times more frequently than other single nucleotide substitutions in primate genomes, and that they exhibit substantial regional variation. Patterns of CpG rate variation are consistent with differences in methylation level and susceptibility to subsequent deamination. In particular, we propose a “distance-decaying” hypothesis, positing that due to the molecular mechanism of a CpG substitution, rates are correlated with the stability of double-stranded DNA surrounding each CpG dinucleotide, and the effect of local DNA stability may decrease with distance from the CpG dinucleotide.

Consistent with our “distance-decaying” hypothesis, rates of CpG substitution are strongly (negatively) correlated with regional G+C content. The influence of G+C content decays as the distance from the target CpG site increases. We estimate that the influence of local G+C content extends up to 1,500∼2,000 bps centered on each CpG site. We also show that the distance-decaying relationship persisted when we controlled for the effect of long-range homogeneity of nucleotide composition. GpC sites, in contrast, do not exhibit such “distance-decaying” relationship. Our results highlight an example of the distinctive properties of methylation-dependent substitutions versus substitutions mostly arising from errors during DNA replication. Furthermore, the negative relationship between G+C content and CpG rates may provide an explanation for the observation that GC-rich SINEs show lower CpG rates than other repetitive elements.

## Author Summary

Mutations are raw materials of evolution. Earlier studies have shown that mutations occur at different frequencies in different genomic regions. By investigating the patterns and causes of such “regional” variation of mutations, we can better understand the mechanisms of underlying mutagenesis. In the human and other mammalian genomes, the most common type of mutation is caused by DNA methylation, which targets cytosines followed by guanine (CpG dinucleotides). Methylated cytosines are then subject to spontaneous deamination, which will cause a C to T (or G to A) transition (CpG substitution). Because this mutational process is unique to CpG substitutions, we reasoned that they might show different patterns of variability from other substitutions. Using high quality genomic sequences from primates and by separately analyzing variability of CpG substitutions and other substitutions, we demonstrate that CpG substitutions occur approximately 15 times more frequently than other substitutions, and show a distinctive pattern of regional variability. Particularly, we propose and provide evidence that because the deamination step requires temporary strand separation, G+C composition near 1,500–2,000 bps each direction from a target CpG affects the probability of a CpG substitution. Incorporating the difference in CpG and other substitutions discovered in this study will help build more realistic evolutionary models.

## Introduction

Elucidating causes of regional variation of substitution rates is a prominent topic in molecular evolutionary studies [1]–[6]. While most studies consider all types of point mutations together, different types of mutations arise from distinctive molecular processes. In this paper, we contrasted regional variations of two types of mutations that are prevalent in mammalian genomes according to their molecular origins.

The first type of point mutations we consider are transitions at CpG dinucleotides, which are caused primarily by methylation of cytosine [7],[8]. Methylation followed by deamination causes a C to T transition (or G to A transition in the complementary strand) at a CpG dinucleotide. We refer to C to T (or G to A) substitutions at a CpG site as “CpG substitutions”. These are the most frequent point mutation in the human genome [2],[9], and often the basis of human genetic disease [10],[11]. In contrast, most other point mutations are believed to occur from errors in DNA replication [12].

Here we show that CpG substitutions occur over one order of magnitude more frequently than other substitutions in primate genomes, and that they exhibit substantial regional variability. We investigated the causes of regional variability of CpG rates. We pay a particular attention to the role of regional sequence context, based upon the differences in mutational mechanisms.

In particular, efficiency of the deamination step may affect regional variation of CpG substitutions [13]. A prerequisite for the deamination process is the insertion of a water molecule between the DNA strands, via temporary *melting* or strand separation, which requires thermodynamic energy [13]–[15]. Bonds between G and C nucleotides require more thermodynamic energy to break, compared with A and T nucleotides [16]. Therefore, the substitution rate at CpG dinucleotides may be negatively correlated with GC content (defined as the percentage of G and C nucleotides). There are conflicting conclusions on this relationship [17]–[19].

Furthermore, it is unlikely that the G+C content of the whole sequence segment affects the probability of a CpG site to mutate. Rather, the effect of G+C content on CpG substitution may be confined to sites that are nearby the CpG site. In other words, the effect of sequence context on a CpG substitution is likely to be *local*. Moreover, we predict this effect will decay as the distance to the target CpG site increases. Therefore, we predict that G+C content has a strong *distance-decaying* influence on the CpG substitution rate, because only *local DNA melting* is required for deamination to occur. We present evidence that is consistent with our *distance-decaying local GC influence hypothesis*.

In addition to our main results on the distance-decaying relationship, we discuss other causative factors of regional heterogeneity of CpG substitution rates. These include differential methylation of some transposable elements and potential variation of mismatch repair efficiency. The factors causing regional CpG substitution rate variation outlined in this paper are important in the study of genome evolution, and in the inference of phylogenetic histories. Our work also highlights the distinct properties of mutations that are dependent on DNA methylation as opposed to those mainly caused by errors during replication.

## Results

### CpG Substitution Rate Exhibits Substantial Regional Variation

We analyzed approximately 38 million orthologous sites from human, chimpanzee, and baboon obtained by aligning genomic DNA segments from these species. We used only high quality Bacterial Artificial Chromosome (BAC) based sequences in all of our analyses. Sequences that resulted from low-coverage whole genome shotgun sequencing projects, which may harbor errors in sequencing and assembly, were not used. We then extracted only non-coding sequences (see Methods) to analyze patterns of substitution rate variation free from the effect of natural selection. Our final non-coding data set included approximately ∼14.7 million aligned sites from 17 chromosomes.

We used a parsimony method [19]–[23] to identify sites that have been part of a CpG dinucleotide in the recent past (“CpG sites”). In addition, using the same method, we identified sites that have been part of a GpC dinucleotide (“GpC sites”, see the Methods section for details). Because GpC dinucleotides consist of the same bases (C and G) as CpG dinucleotides, while not involved in DNA methylation [24], they are often used as a dinucleotide control for CpG sites [13],[17],[25]. We also analyzed all sites that have not been a part of a CpG dinucleotide during the given evolutionary timescale (“non-CpG sites”, [22]). Note that GpC sites are a subset of non-CpG sites. Results from non-CpG sites were similar to that from GpC sites (see below).

It is important to note that a great majority of CpG sites in certain regions, called CpG islands, of mammalian genomes are typically hypo-methylated [26] and hence do not undergo methylation-origin mutation process. Therefore, it is crucial to exclude CpG islands from our analyses. We used similar but slightly more stringent criteria to those proposed by Takai and Jones [27], a widely used method, to identify and exclude CpG islands in our data (see Methods). We chose to use a shorter length cutoff than the conventional length as proposed in an earlier study [28], so that our method will be conservative in terms of masking true CpG islands.

Table 1 describes the numbers of CG->TA transition substitutions in CpG and GpC sites in our dataset. Note that even though there are over an order more GpC sites than CpG sites in our data, the total numbers of CpG and GpC transition substitutions are similar. This observation confirms that CpG substitutions occur much more frequently than other types of substitutions in the human genome [2],[29],[30]. For simplicity, we refer to the proportion of the number of transition substitutions to the total number of (CpG or GpC) sites as the “rate of substitution” in the rest of our paper, which implies that the unit of timescale is since the divergence of the genomes of humans and chimpanzees. The rate of CpG substitution in intergenic regions and introns are 14.29±0.4%, and 13.88±0.5%, respectively. The rate of GpC substitution in intergenic regions and introns are 1.12±0.037% and 0.98±0.043%, respectively.

**Table 1:** Description of the Dataset.

Type of site		Intergenic		Introns	
		No. sites	No. substitutions*	No. sites	No. substitutions*
CpG	Repetitive	11257	1761	6941	985
	Non-repetitive	11277	1460	7631	1038
	Total	22534	3221	14572	2023
GpC	Repetitive	154760	1844	84577	827
	Non-repetitive	152695	1619	111815	1106
	Total	307455	3463	196392	1933

The number of CpG and GpC sites identified in our dataset and the number of CG->TA transition substitution in these sites.

*: CG->TA transition substitutions only. The substitutions in humans and chimpanzees were pooled.

See Methods section for definition of sites. There were 8,971,241 and 5,767,731 aligned sites in intergenic regions and introns, respectively.

**Figure 1:**
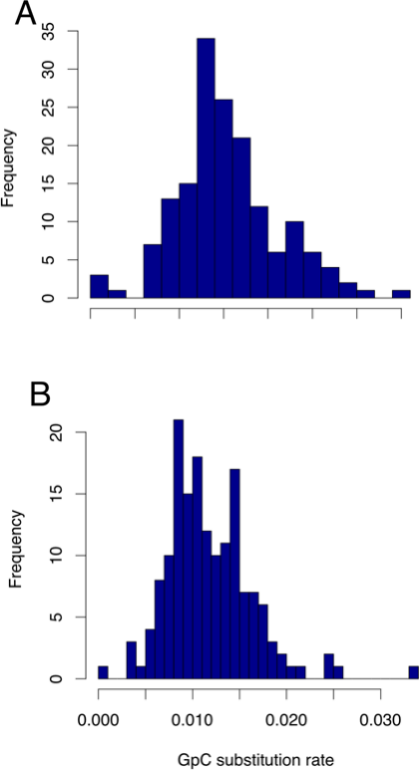
Histogram of the rate of CG->TA substitutions in non-coding regions. The rate of CG->TA transitions in CpG sites (A), and GpC sites (B) in 50 kb segments of non-coding regions having at least 10,000 aligned sites. Variation of CpG substitution rate among non-coding regions is significantly greater than that expected under a uniform substitution rate model. A similar result was obtained for GpC sites (see text).

When all single nucleotide substitutions are considered, their rates vary substantially among different regions, more than expected solely from stochastic effects [2]. To examine regional variation of CpG substitution rates, we plotted the rate of CpG substitution in 50 kb segments of non-coding regions with at least 10 kb aligned sites (Figure 1A). The mean CpG substitution rate in these segments is 15.3%. The observed standard deviation is 6.3%, which is significantly greater than the standard deviation expected under a model that assumes uniform CpG substitution rate in all the segments (95% confidence interval [CI] 4.2%–5.8%; see Methods). As expected, GpC sites and non-CpG sites also exhibited substantial variation (Figure 1B). These results remained the same when we changed the size of the windows examined.

The rates of CpG substitution and those of non-CpG substitutions are significantly correlated in our sample (Pearson correlation coefficient *ρ* = 0.32; *P*<0.001). Because CpG substitution rate did not follow a normal distribution, we also analyzed log-transformed data and obtained a similar result (*ρ_tr_* = 0.31; *P*<0.001).

### CpG Substitution Rate in Non-Coding Regions of Primate Genomes Is Negatively Correlated with G+C Content

**Figure 2:**
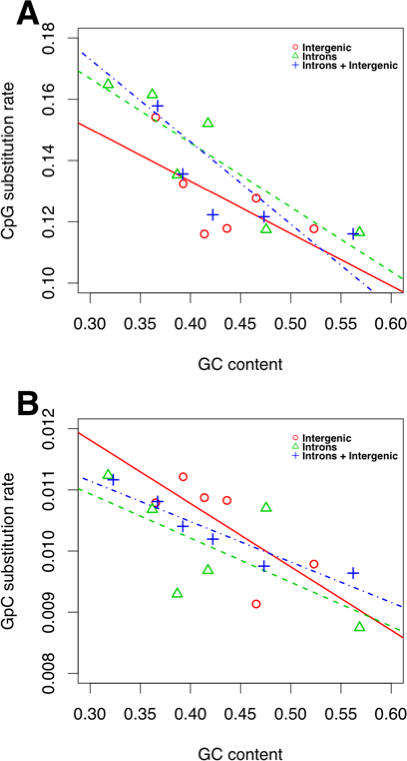
Negative correlation between the rate of CG->TA substitution and G+C content of non-coding regions. (A) Non-coding regions (intergenic and introns) were partitioned into six equal-sized bins based on their G+C contents. The rates of CG->TA substitutions in CpG sites of these bins are negatively correlated with their G+C contents. The negative relationship holds when introns were analyzed separately. In case of intergenic regions the relationship was not significant. Nevertheless, we found a negative trend. (B) GpC substitution rates in non-coding regions exhibited a negative relationship with G+C contents. When divided into intergenic regions and introns, however, the relationships were not significant, although there was a clear negative trend. Refer text for r*^2^* values and *P*- values.

Earlier studies reported conflicting results on the relationship between the G+C content and the CpG substitution rate of a genomic region. Some studies have proposed a negative relationship [13],[17],[18] while others observed no correlation [19]. We analyzed this relationship by dividing the non-coding regions into 6 equal size bins based on their G+C content and plotted the rate of CG->TA substitution at CpG sites in each bin against its average G+C content (Figure 2A). To avoid the effect of variation in methylation efficiency, sites in transposable elements were excluded from analyses in this and the following section.

We observed a significant negative correlation between the G+C content of the non-coding regions and the CpG substitution rate (Figure 2A; r*^2^* = 0.642, *P* = 0.032). When the CpG data was partitioned into introns and intergenic regions, the negative relationship with G+C content was significant in introns (r*^2^* = 0.71, *P* = 0.021), but not in intergenic regions although there was a clear negative trend (r*^2^* = 0.29, *P* = 0.15). The average length of the intron segments is 5.5 kb and of the intergenic segments is 119 kb.

We also investigated rates of CG->TA substitutions at GpC sites, as a control for sites that are not affected by DNA methylation [24]. We observed a negative correlation with the G+C content of the non-coding regions (Figure 2B; r*^2^* = 0.86, *P* = 0.004). When the GpC data was partitioned into introns and intergenic regions, the relationships were not significant [r*^2^* = 0.313 (*P* = 0.14), r*^2^* = 0.341 (*P* = 0.10) for introns and intergenic regions, respectively]. Nevertheless, we observed negative trends in both introns and intergenic regions (Figure 2B).

### Distance-Decaying Relationship between CpG Substitution Rate and G+C Content

The negative relationship between CpG substitution rate and G+C content (see above) is consistent with the thermodynamic requirement during deamination process (see Introduction). However, given that transition rates at other sites (such as GpC sites) also exhibit negative relationship with G+C content (see above) and that thermodynamic hypothesis does not necessarily explain long-range effect of G+C content on CpG substitution rates, we proposed the “distance-decaying” hypothesis.

We now present our main results on the distance-decaying influence of G+C content on rates of CpG substitution. We performed a sliding window analysis using a window size of 200 bps and a step size of 25 bps (partially overlapping windows) to analyze the relationship between G+C content at varying distances from the CpG site and the rate of CpG substitution. Because the average length of the introns in our dataset was only ∼5.5 kb [small as compared to the average length of intergenic regions (119 kb)], a large proportion of CpG sites in introns will have a large portion of their sliding windows lie in exons (the sliding windows extended up to 5 kb around each CpG site, see below). Therefore, we used only intergenic CpG sites in this analysis, to minimize the effects of constraints related to coding for amino acids and natural selection. When we included a subset of intronic CpG sites that are at least 3 kb away from an exon in our analyses, the results were the same (Figure S5). Thus, the observed pattern is a common feature of non-coding regions in primate genomes.

**Figure 3:**
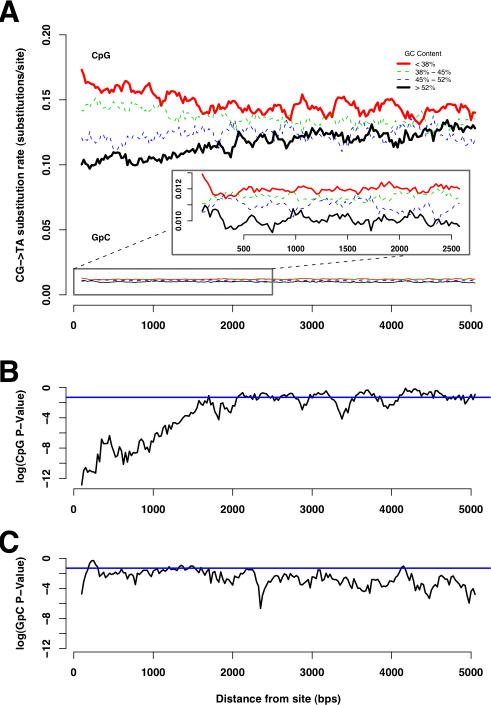
Sliding window analysis of the relationship between CpG substitution rate and G+C content of windows. (A) At each distance (along the X-axis), CpG sites were divided into four bins based on G+C content at that distance from the site (as measured from the G+C content of the 200 bps window centered at that distance; G+C<38: red curve, 38< = G+C<45%: green curve, 45< = G+C<52%: blue curve, G+C>52%: black curve). The proportion of CpG sites mutated in each of these bins is plotted as a function of distance from the site. At distances closer to the CpG site, the rate of substitution in high local-GC bins (black curve) is clearly lower compared to that of low local-GC bins (red curve). This relationship progressively declines as we move farther away from the site, suggesting a distance-decaying relationship between G+C content and CpG substitution rate. In case of GpC sites, we do not observe a distance-decaying effect (see inset). (B) Results of the chi-square test for the independence of the rate of CpG substitution and the G+C content of the windows at each distance, in log scale. The blue line indicates the *P*-value cutoff of 0.05 [or log_10_ (*P*-value) = −1.30]. The *P*-values are very low at distances close to the CpG site, and progressively become larger as the distance from the CpG site increases (distance-decaying effect). The rate of CpG substitution becomes independent of the G+C content [log_10_ (*P*-value)>−1.30] after ∼2,000 bps from the CpG. (C): Results of the chi-square test for the independence of the rate of GpC substitution and the G+C content of the windows at each distance, in log scale. Again, the blue line indicates the *P*-value cutoff of 0.05 (or log_10_ (*P*-value) = −1.30). The rate of GpC substitution becomes independent of the G+C content [log_10_ (*P*-value)>−1.30] at a distance very close to the GpC site, and no distance-decaying effect was observed.

At each distance, we binned CpG sites with similar G+C content (as measured from the 200 bps sliding window) at that distance from the site. The cutoffs used for binning were <38%, 38%–45%, 45%–52%, >52%. These cut-offs divided the data into similar bin size and also roughly corresponded to the traditional definition of isochores [31]. For each distance *i*, and each G+C content bin, we then calculated the rate of CpG substitution (as the proportion of CpG sites that are mutated). More formally, for each G+C content bin *B*, we considered:
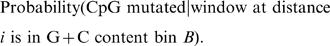



The results of this analysis are shown in Figure 3A. We observed a clear effect of G+C content of windows close (less than 2,000 bps in each direction) to the target CpG site. Higher G+C content in the window lowers the CpG substitution rate (as expected from the negative relationship between G+C content and CpG rate, reported in the previous section). This effect is the most pronounced at distances very close to the target CpG site. For example, ∼17% of the CpG sites exhibiting low G+C content (GC<38%; red color curve in Figure 3A) at distance 100 bps are mutated, while the same measure for CpG sites with high G+C content (GC>52%; black color curve in Figure 3A) at distance 100 bps is ∼11%. As we move farther away from the CpG site (Figure 3A; left to right along the X axis), the rate of CpG substitution in the low G+C bin (red color curve) and the high G+C bin (black color curve) progressively become closer to each other, displaying the distance-decaying effect of G+C content on the rate of CpG substitution. This effect appears to vanish at around 2,000 bps from the CpG site (i.e., 4,000 bps around the CpG site).

We used a chi-square test to determine whether the rate of CpG substitution is dependent on G+C content, at each distance point (Figure 3B). The distance-decaying relationship is clear when we observe the *P*-value obtained at each distance. We observe very low *P*-values (*P*<<0.05 or log_10_ (*P*-value)<<−1.30; which means that the rate of CpG substitution is highly dependent on G+C content) at distances close to the CpG site. As we move farther from the CpG site, the *P*-value increases progressively, becoming insignificant at ∼2,000 bps, consistent with the results observed in Figure 3A.

We obtained similar results with different overlapping window sizes [for example, window size of 25 bps and step size of 5 bps (Figure S1)], and when 6 bins were used instead of 4 bins (however, using 6 bins decreased the sample sizes and thus increased the fluctuations in the figures). Similar results were obtained with non-overlapping windows (results not shown).

Note that even though there is a distance-decaying relationship between CpG substitution rate and G+C content in immediate neighboring sites, the CpG substitution rate in each GC category differ, although not significantly, from each other even after 2,000 bps. In particular, the CpG substitution rate in low GC bins (red curve in Figure 3A) did not converge with the CpG substitution rate in high GC bins (black curve in Figure 3A) even after 2,000 bps. In the following section, we show that this may be a consequence of long-range (global) nucleotide composition.

As a control for other dinucleotide sites, we performed the sliding window analysis using GpC sites. In contrast to the case of CpG sites, we do not observe a distance-decaying relationship between G+C content and the rate of GpC substitution (GpC curves in Figure 3A). The *P*-values for testing the independence of the substitution rate at GpC sites and the G+C content was significant at distances >2,000 bps, suggesting a negative relationship between GpC substitution rate and long-range G+C content (see the following section for more details).

### The Distance-Decaying Relationship Persists after Correcting for Variation in Global G+C Content

Although the distance-decaying negative relationship between CpG substitution rate and G+C content subsides at ∼2,000 bps (i.e., 4,000 bps around the CpG site; Fig. 3B), the rate of CpG substitution in low G+C content bin continued to remain higher than that of high G+C content bin (Figure 3A). GpC rates also differed between high and low G+C contents (Figure 3A and 3C).

Indeed, both CpG and GpC rates were significantly negatively correlated with G+C contents of 5 kb, 20 kb, and 100 kb blocks around each dinucleotide (results not shown). Also, the G+C contents of 5 kb, 20 kb, and 100 kb blocks are all positively correlated (results not shown), presumably because of the isochore structure of primate genomes. In the remainder of this paper, we refer to the G+C content of 100 kb segments around each CpG site as *GC_global_*, and the negative relationship between *GC_global_* and CpG substitution rate as a “*global effect*”. We tested if the distance-decaying effect of local (<4,000 bps) G+C content exists even after controlling for global GC effect (by removing variation in *GC_global_*) by the following two analyses.

In the first analysis, we performed the aforementioned sliding window experiment with the G+C content of the windows normalized by the G+C content of the 100 kb segment flanking the CpG site. Precisely, we define the normalized G+C content of a window, denoted *GC_norm_*, as:
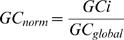
where GC*i* denotes the G+C content of the window at distance *i* from the CpG site and *GC_global_* denotes the G+C content of the 100 kb region surrounding the CpG site. The value *GC_norm_* represents the “relative” G+C content of the *i*-th window with respect to the G+C content of 100 kb segment within which it belongs.

**Figure 4:**
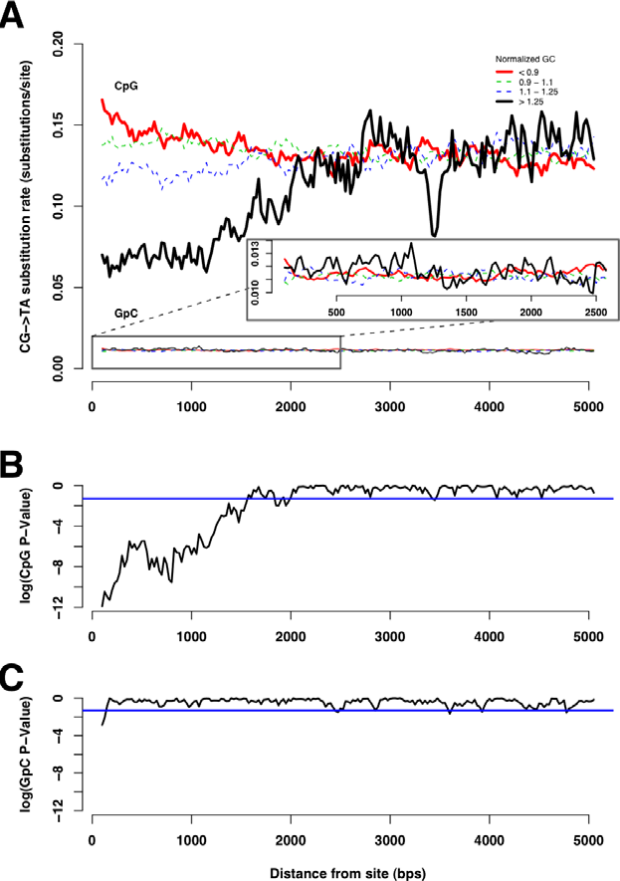
Sliding window analysis of the relationship between CpG substitution rate and normalized G+C content. The same experiment as in Figure 3 with the G+C content of each window normalized with respect to GC*_global_* (removing global effect). (A) The distance-decaying effect of G+C content on the rate of CpG substitution persists even after removing the global effect. In case of GpC substitutions, there was no distance-decaying effect. (B) Results of the chi-square test for the independence of the rate of CpG substitution and the G+C content of the windows. The blue line indicates log_10_ (*P*-value) = −1.30. The distance-decaying effect subsided after ∼1,500 bps. (C) Results of the same experiment as in (B), but for GpC sites. There is no distance-decaying effect, as expected.

Figure 4 shows the results of the sliding window analysis, where the G+C contents of the 200 bps windows (GC*i*) are normalized. The *GC_norm_* cutoffs we used were <0.9, 0.9–1.1, 1.1–1.25, >1.25. These cutoffs were chosen because they divided the data into approximately equal size bins. Even when variation in *GC_global_* was removed, we found the same decaying effect of local G+C content on the rate of CpG substitution (CpG curves in Figure 4A, and 4B). The G+C content of windows closer to the CpG site affected the rate of substitution more compared to that of windows farther away from the CpG sites (CpG curves in Figure 4A and 4B). In contrast to the CpG curves in Figure 3A, the CpG curves in Figure 4A converged, suggesting that the non-convergence in Figure 3A is in fact caused by the global effect. The curves converged at a distance of ∼1,500 bps, which is slightly lower than that observed in Figure 3A. In the case of GpC sites, as expected, there was no distance-decaying relationship and the curves converged at a distance very close to the site (GpC curves in Figure 4A and 4C).

In our second analysis, we first focused on the distribution of *GC_global_*. *GC_global_* exhibits a bimodal distribution with means of ∼39% and ∼48%, respectively (Figure S2A). A similar distribution of G+C content was observed in case of 100 kb segments surrounding GpC sites (Figure S2B). These bimodal distributions occur because the number of CpG sites and GpC sites are expected to increase with the G+C content of the non-coding region. To reduce variation in *GC_global_*, we analyzed these two distributions (which we call as low- *GC_global_* and high-*GC_global_* regions) separately.

We performed the sliding window analysis to test for the distance-decaying effect of local G+C content in low-*GC_global_* and high-*GC_global_* regions (without normalizing). In the case of CpG sites in low-*GC_global_* regions (Figure S3A and S3B), we obtained similar results as in Figure 4A. In the case of CpG sites in high-*GC_global_* regions, there were large fluctuations in the trend, especially in the low local-GC bins (Figure S4A; red CpG curve). These fluctuations are caused by the reduced sample size in this bin (data not shown). Nevertheless, the distance-decaying effect is clear when we consider the curves of high and low local-G+C content bins (black and red curves CpG, respectively, in Figure S4A). Consistent with the results from Figure 4, the distance from the CpG site at which the curves converged was ∼1,500 bps (Figure S3A). Again, GpC sites did not exhibit any distance-decaying effect (Figures S3A, S3C, S4A, S4C).

Thus, even after removing the global effect, the distance-decaying effect of local G+C content on CpG substitution rate persists, and it subsides after approximately 1,500 bps in each direction from each CpG site. These results were robust to using the flanking 5 kb or 20 kb window for the normalization, different window sizes, and non-overlapping windows (results not shown).

### Other Factors That Cause Variation of CpG Substitution Rates

The previous sections analyzed the effect of the efficiency of deamination step on CpG substitution rates. In this section we briefly discuss other factors that cause regional heterogeneity of CpG substitution rates.

For this purpose we consider that a CpG substitution occurs in a three-step process (this is a simplification, but for our purposes this view suffices). First, DNA methylation in mammals (and other warm-blooded vertebrates) occurs specifically at the cytosine bases of CpG dinucleotides. Second, the methylated cytosine undergoes spontaneous deamination. Deaminated methyl-cytosine is identical to thymine, creating a C to T mismatch at the CpG dinucleotide. Third, if this mismatch is left un-repaired, it causes a C to T transition (or a G to A transition in the complementary strand) in the next replication cycle. Based upon such molecular mechanism of the origin of CpG substitution, their rates may vary due to differences in (i) levels of germline methylation, (ii) efficiency of deamination of a methylated cytosine, and (iii) repair efficiency of a transition that occurred via deamination of a methylated cytosine.

A strong support for the first cause, differential methylation, is the observation that transposable elements have generally higher rates of CpG substitutions [19]. Proliferation of transposable elements can potentially impose several deleterious effects on a genome, because insertion of transposable elements can disrupt gene regulation, or cause deleterious recombination events, translocations and other rearrangements [32]. Hence, some organisms may use DNA methylation as a defense mechanism against the proliferation of transposable elements [19],[33].

In our data, we found that transposable elements as a whole exhibit ∼14% higher CpG substitution rate as compared to that of non-repetitive regions (see Text S1). Overall, there is a significant positive correlation between CpG rates and the proportion of transposable elements in a non-coding segment (*ρ = *0.142; *P*<0.001 and *ρ_tr_ = *0.199; *P*<0.001), consistent with the idea that transposable elements have higher CpG substitution rates as a consequence of increased methylation.

Interestingly, when transposable elements were divided into different classes of elements (LTR, LINE, SINE and DNA elements), SINEs showed similar level of CpG rates to non-repetitive sequences. This was also observed by Meunier et al. [19], who proposed that it might be a consequence of the complex methylation pattern of SINEs in germline cells [34]. However, another possibility is that SINEs show lower CpG rate because their G+C content is in general higher compared to other classes of transposable elements, in light of our finding that there is a negative relationship between CpG substitution rates and G+C contents (see above). SINEs have the highest G+C content among the classes of transposable elements considered in our analyses (50.7%, while 36.6%, 44.0%, 36.4%, for LINEs, LTRs and DNA transposons). This mechanism can at least partially explain why SINEs have lower CpG substitution rates. Because a major proportion of CpG sites in the transposable elements of introns were contributed by SINEs, the rate of CpG substitution in intronic transposable elements is comparable to that of intronic non-repetitive regions (see Text S1).

If the efficiency of the third step, namely mismatch repair, affects regional variation of CpG substitution rates, we expect to see a significant negative correlation between recombination rates and CpG substitution rates. Indeed, we did find a significant negative correlation between recombination rates and the rates of CpG transition substitution (Spearman’s *ρ* = −0.165, *P*<0.001). This can be due to the direct effect of biased gene conversion acting on mutations at CpG sites, repairing the TG mismatches (caused by deamination of methyl-cytosine) to correct CG base pairs, the efficiency of which depends on the rate of recombination. Alternatively, biased gene conversion may increase G+C content, which in turn reduces the intensity of DNA melting, leading to a lower rate of substitution at CpG sites. In the first scenario, a significant correlation between CpG substitution rate and recombination rate is expected independent of the effect of G+C content. In the second scenario, the effect of recombination may disappear after correcting for G+C content. In our case, there was no significant correlation after removing the effect of G+C content using partial correlation analysis (*ρ*_recombination, CpG rate | GC_ = −0.03, *P*>0.05). However, this question needs to be revisited with more data, most certainly when a higher-quality whole genome alignment of human, chimpanzee, and rhesus macaque becomes available.

## Discussion

Even though the prevalence and importance of CpG substitutions is long recognized [8], the causes and patterns of their regional variation have been relatively little explored. In this study, we analyzed a large amount of high-quality genome sequence data (based upon BAC-clones) to provide a better understanding on the patterns and causes of variation of CpG substitution rates in primate genomes.

The negative relationship between CpG substitution rate and G+C content is consistent with the idea that variations in the efficiency of the deamination process [13]–[15],[17] cause regional variation of CpG substitution rates. On the other hand, biased gene conversion can also explain this relationship. Even though we cannot distinguish the relative contributions of these two processes by our analysis, the distance-decaying relationship between G+C content and CpG substitution rate strengthens the former hypothesis.

Our distance-decaying hypothesis is contradictory to conclusions drawn by Fryxell and Moon [17] and Zhao and Jiang [25]. Fryxell and Moon [17] conclude that G+C content of short and long segments surrounding each CpG site has similar degrees of correlations with the rate of CpG substitutions; this is clearly counter to the distance-decaying influence hypothesis. However, their analysis was based on a considerably smaller dataset (a total of 4437 CpG and GpC sites, as compared to 497467 sites used in this study; see Table 1), and only two length scales (564 bps and 163 kb) were analyzed. In another study, Zhao and Jiang [25] analyzed a larger human SNP dataset (292216 CpG and GpC sites) and observed that the absolute value of the slope of the relationship between log (CpG substitution rate) and G+C content *increased* with the length over which G+C content was measured (segment lengths of 101 bps to 1,001 bps around the CpG site). Their results are counter to the thermodynamic mechanisms which were first suggested by Fryxell and Zuckerkandl [13] and now drive our hypothesis. The discrepancy is likely due to the different method and dataset used by Zhao and Jiang [25], as compared to our study (see Text S1 for details). Our results in this vein support the thermodynamic mechanism.

Our analyses (Figure 3 and Figure 4) show that the CpG substitution rate has a significantly stronger relationship with the *local* G+C content. The influence of the G+C content decays with the distance to the target CpG site, subsiding at around 1,500–2,000 bps for each direction. There are at least two immediate questions raised by our analysis. First, what are the causes of the local distance-decaying relationship, which extends up to 1,500–2,000 bps? Given the absence of evidence for the effect of transcription coupled repair or transcription induced deamination (only intergenic regions were analyzed), or change in mutational biases over time [9], we propose that the distance-decaying relationship may be a consequence of the poorly understood mechanism of DNA melting [35], which is required for the deamination process.

The second question is whether there is both global and local effect of G+C content on CpG substitution rate. We raise this question because it is possible that only the local G+C content may affect the CpG substitution rate, and the global effect is a consequence of the so-called “isochore” structure, which causes a positive correlation between local G+C content and global G+C content [31],[36],[37]. Although the answer to this question is still unclear and more direct experiments are required, the convergence of CpG curves when the data is divided into low-GC*_global_* and high-GC*_global_* distributions (For example, Figure S2A and S2B) despite the fact that there is some variation in GC*_global_* even within these distributions (Figure S1A) suggests that GC*_global_* may not have a direct effect on the rate of CpG substitution.

We found a negative relationship between GpC substitution rate and G+C content (Figure 2B). As suggested by Fryxell and Moon [17], this relationship can be explained by biased gene conversion [38] or by regional differences in the deamination of unmethylated cytosines (an underlying mutational bias). In the absence of a distance-decaying relationship between GpC substitution rate and local G+C content (Figure 3A and Figure 4A), which is expected if deamination plays a major role in causing GpC substitution rate variation, it seems more likely that biased gene conversion is the cause of the negative relationship between G+C content and GpC substitution rate.

Our results demonstrate the significance of the distinctive properties exhibited by methylation dependent substitutions versus substitutions caused by other molecular mechanisms. Models of molecular evolution and methods of phylogenetic inference, especially those concerned with mammalian and bird genomes (which have high rates of CpG substitutions), may benefit by considering the local effect of G+C content on CpG substitution rates (e.g., [5],[18],[30]).

## Methods

### General Approach

We can use a parsimony method [19]–[23] to infer CpG sites and determine the substitution rate in humans and chimpanzees using baboon as an outgroup. However, we cannot distinguish substitutions caused by methylation followed by deamination versus replication errors. In primates, CpG substitutions are markedly more frequent than other single nucleotide substitutions [2],[21],[29]. Therefore we assumed methylation followed by deamination is the primary cause of all CpG substitutions in these genomes.

### Human, Chimpanzee and Baboon Sequences

We analyzed ∼38 Mbps of sequence data obtained from two different sources; 22 Mbps from sequences orthologous to the human chromosome 7 sequenced by the NISC comparative sequencing group, and ∼15 Mbps obtained from other chromosomes from database mining.

#### Sequence data from chromosome 7

We analyzed ∼22 Mbps from human chromosome 7. The sequences are the same as that in Dataset 1 of [39]; however, we made our analysis more stringent by removing introns that are alternatively spliced (see below). Chimpanzee and baboon BAC clones orthologous to the regions in human chromosome 7 were isolated and sequenced as described in [40] and [41].

#### Sequence data from other chromosomes

For other chromosomes, we mined sequence data from GenBank [42]. Briefly, we downloaded all the baboon and chimpanzee BAC clones available as of March 2006 from GenBank and then used Blastz [43] to determine the high scoring matching segments. Then, we used a pipeline of programs to perform chaining and netting, as described in Kent et al. [44], to establish orthology. A detailed description of the procedure used is presented in Text S1.

### Sequence Annotation and Alignment

Sequences orthologous to human chromosome 7 were aligned using the Threaded Blockset Aligner program [45]. The “best chains” (Text S1) from other chromosomes were aligned using the Multiz program [45]. Non-coding regions (introns and intergenic regions) were identified using gene annotations included in the Known Genes and Ensembl Genes tables of the *hg*17 assembly of the human genome at UCSC Genome Browser [46]. Intergenic and intronic sequences likely to be selectively constrained [the 5′ and 3′ untranslated regions, first introns and small (<250 bps) introns or intergenic intervals] were excluded. In addition, we excluded alternatively spliced introns based on gene annotations from the UCSC genome browser. In particular, if the span of an intron is different in different transcripts of the same gene, it was removed. The above methods yielded ∼14.7 Mbps of aligned sites in the non-coding regions of the human genome (Table 1). Recombination rates were obtained from [47].

### Identification of CpG Islands

CpG islands are regions of the genome where a majority of CpG sites are not methylated. Because methylation of the cytosine in CpG dinucleotides is a prerequisite for the CG->TA mutation to occur (see Introduction), it is crucial for our analyses to exclude CpG sites in CpG islands. Takai and Jones [27] proposed that a good definition of CpG islands is- a region of the genome with (a) G+C content >55%, (b) length >500 bps and (c) observed/expected proportion of CpG dinucleotides (OE) >0.65. The condition length >500 bps was used by Takai and Jones to eliminate the possibility of falsely calling a CpG rich regions generally associated with Alu elements as CpG islands. However, there may be some CpG islands less that 500 bps in length.

In this study, we changed the length constraint to >200 bps to err on the side of caution to safely eliminate most of the CpG islands. We masked out CpG islands identified using the algorithm by Takai and Jones [27] with parameters G+C content >55%, OE>0.65, length >200.

### Identification of CpG Sites

To identify CpG sites, we used a parsimony method. Specifically, CpG sites are the middle base of the sites having the following human/chimpanzee/baboon patterns: XNG/XCG/XCG or XCG/XNG/XCG, where X is any nucleotide except G. Given the evolutionary distances considered here, in spite of the hypermutability of CpG sites, such a definition is shown to be quite accurate by a simulation study in Meunier and Duret [22]. GpC sites are sites having the following human/chimpanzee/baboon pattern GNY/GCY/GCY or GCY/GNY/GCY, where Y is any nucleotide except G. The restrictions on X and Y were imposed to avoid overlapping CpG and GpC sites. Non-CpG sites are defined as sites not preceded by a C and not followed by a G. Sites following the complementary patterns of the above definitions were also considered as CpG and GpC and non-CpG sites.

### Substitution Rate Estimates and Statistical Tests

To obtain better estimates of substitution rates, we pooled together substitutions in human and chimpanzee lineages. The rate of substitutions for a particular class (CpG or GpC) of site was estimated by dividing the number of substitutions by the total number of sites in that class.

### Simulation of Uniform Rate Model

To test if the observed variation in CpG substitution rate in the 50 kb segments is greater than that expected under a uniform substitution rate model, we performed the following simulation. For each segment (*s*), we kept the number of CpG sites (*n_s_*) the same as that observed in our data and sampled the number of substitutions from the binomial distribution *b(n_s_,p)*, where *p* is the probability of observing a CpG substitution. *p* was kept constant across segments (0.153 in the case of CpG sites). We simulated 1,000 replicates and found the standard deviation of CpG substitution rate among the segments in each replicate. The range between 2.5% quantile and 97.5% quantile was taken as the 95% confidence interval of the standard deviation under uniform substitution rate model. A similar simulation was also performed for GpC and non-CpG sites.

## Supporting Information

Text S1 Mutations of different molecular origins exhibit contrasting patterns of regional substitution rate variation.
(0.05 MB DOC)

Figure S1 Sliding window analysis of the relationship between CpG substitution rate and normalized G+C content. The same experiment as in Figure 3 with window size 25 and step size 5. (A) The distance decaying effect of G+C content on the rate of CpG substitution persists even with a smaller window size of 25 bps (as compared window size of 200 bps in Figure 3). In the case of GpC sites, there was no distance decaying effect. (B) Results of the chi-square test for the independence of the rate of CpG substitution and the G+C content of the windows. The blue line indicates log10 (P-value) = −1.30. The distance decaying effect subsided after ∼2,000 bps. (C) Results of the same experiment as in (B), but for GpC sites. There is no distance-decaying effect.
(4.36 MB TIF)

Figure S2 The distribution of GC content in 100 kb segments around CpG and GpC sites. (A) The G+C content of 100 kb segments around CpG sites GC_global_ followed a bimodal distribution with means 39% and 48%, respectively. The red line indicates GC_global_ = 43%, which was used as the cutoff to differentiate between low- GC_global_ and high-GC_global_ regions. (B) G+C content of 100 kb segments around GpC sites also exhibited a bimodal distribution, with approximately the same means as those of GC_global_. The red line marks G+C content of 43%.
(0.29 MB TIF)

Figure S3 Distance decaying relationship between G+C content and CpG substitution rate in low- GC_global_ regions. Same analysis as in Figure 3 in the paper with CpG (and GpC) sites with G+C content of 100 kb segments around them less than 43%. (A) The distance-decaying effect of local G+C content on the rate of CpG substitutions was apparent, and the curves converged at ∼1,500 bps. In case of GpC, there was no distance-decaying effect. (B) The test for independence of G+C content and the rate of CpG substitutions. The distance-decaying effect was apparent from the gradual increase of P-values with increase in distance. P-Values become insignificant at ∼1,500 bps. (C) The results of the test for independence of G+C content and the rate of CpG substitutions. No distance-decaying effect was observed between GpC substitution rate and G+C content.
(4.36 MB TIF)

Figure S4 Relationship between G+C content and CpG substitution in high-GC_global_ regions. Same analysis as in Figure S2 with CpG (and GpC) sites with G+C content of 100 kb segments around them greater than 43%. (A) The distance-decaying effect for CpG sites was not apparent because of the fluctuations caused by reduced sample size in bins. In case of GpC, there was no distance-decaying effect. (B) The test for dependence of CpG substitution rate and G+C content was insignificant starting at distances close to the CpG site. (C) No distance-decaying effect was observed between GpC substitution rate and G+C content.
(4.36 MB TIF)

Figure S5 Relationship between G+C content and substitution rate when CpG sites from introns were included. Same analysis as in Figure 3 with CpG sites that lie within introns and that are at least 3 kb away from exons included in the data set. The results are similar to that obtained in Figures 3 and 4.
(9.38 MB TIF)
