# Supplementary material for: Mutations of Different Molecular Origins Exhibit Contrasting Patterns of Regional Substitution Rate Variation
Source: PLoS Comput Biol. 2008 Feb 29;4(2):e1000015. doi: 10.1371/journal.pcbi.1000015 (PMC2265638; doi:10.1371/journal.pcbi.1000015)
Supplement: Text S1 — Mutations of different molecular origins exhibit contrasting patterns of regional substitution rate variation. (0.05 MB DOC) [file pcbi.1000015.s001.doc]

**Text S1**

Mutations of different molecular origins exhibit contrasting patterns of regional substitution rate variation

Navin Elango, Seong-Ho Kim, NISC Comparative Sequencing Program, Eric Vigoda, Soojin V. Yi

**Obtaining the human-chimpanzee-baboon triple alignments for sequences mined from GenBank**

We obtained 377 baboon BACs from Genbank (Benson et al. 2006). Blastz (Schwartz et al. 2003) was used to align the baboon BACs to the human genome. The high scoring segment pairs returned by Blastz were converted into PSL format using the lavToPsl program and chained using the axtChain program. Next, the “best chain” for each baboon sequence was obtained using a perl code. To be the best chain of a baboon sequence, a chain must satisfy two conditions: First, it must be the highest scoring chain for that baboon sequence. Second, it must cover at least 60% of the total length of the baboon sequence. We further filtered the best chains such that the score per site is at least 50. There were 274 such chains. These chains were then “netted” against the human genome using the chainPreNet and chainNet programs. The nets were then split based upon the chromosomes and converted into human-baboon alignments (in MAF file format) using the following programs in order netChainSubset (with –splitOnInsert option), chainToAxt, axtToMaf.

A similar procedure was used to obtain human-chimpanzee alignments (the score pre site cutoff for chimpanzee best chains was 80, and there were 922 chains satisfying this condition). The human-chimpanzee and human-baboon alignments were projected on to the human sequence using the mafProject program, converted to human-chimpanzee-baboon triple alignments using the multiz program. Finally the triple alignments were converted into fasta format using the maf2fasta program.

**Higher rates of CpG substitutions in some transposable elements**

In our data, transposable elements had a significantly higher rate of CpG substitutions as compared to non-repetitive regions: the rate of CpG substitution is ~14% higher in transposable elements as compared to non-repetitive regions (15.08 ± 0.5%in transposable elements compared to 13.21 ± 0.4%in non-repetitive regions).

However this difference was only significant in intergenic regions. While there was a 21% increase in the rate of CpG substitution of intergenic transposable elements as compared to intergenic-non-repetitive regions (15.64 ± 0.6% in repetitive-intergenic as compared to 12.94 ± 0.6% in non-repetitive intergenic), rates for intronic transposable elements was 14.19 ± 0.8%, not significantly different from those for non-repetitive intronic regions 13.60 ± 0.7%, *P* > 0.05).

This disparity was caused by at least two factors: first, different classes of repetitive elements exhibit different rates of CpG substitutions, and second, that genic (intronic) and non-genic (intergenic) regions harbor different types of transposable elements. Different types of transposable elements exhibited different rates of CpG substitutions. CpG substitution rates for LTRs, LINEs and DNA transposons 18.3 ± 1.9%, 17.5% ± 1.5% and 16.1% ± 3.5% respectively, significantly greater than rates for non-repetitive regions (13.21 ± 0.4%). SINEs however have a lower rate (13.20 ± 0.8%), similar to that of the non-repetitive regions (the difference in CpG substitution rates among different transposable element classes has previously been observed by Meunier et al. 2005).

Second, SINEs are more enriched in genic, high GC regions (Lander et al. 2001). In our data, SINEs contribute to 71.8% of all CpG sites in the repetitive portions of introns, compared to 56.1% of CpG sites in intergenic repetitive regions. When SINEs were removed from the data, rates in intronic transposable elements were significantly higher than intronic non-repetitive regions (18.22 ± 1.3% versus 13.60 ± 0.7%).

**Previous studies on length effect and CpG substitution rates**

As we discuss in the text, other studies including Fryxell and Moon (2005) and Zhao and Jiang (2007) have investigated length effects on rates of CpG substitutions. In particular, Zhao and Jiang (2007) analyzed 292216 CpG and GpC sites from the Human SNP database (<http://www.ncbi.nlm.nih.gov/projects/SNP/>). Briefly, they used noninsertion/deletion, non-repetitive, biallelic polymorphic sites for which the sequence data of at least 100 nucleotides flanking each side of the site is known. The flanking sequences were then used to map the polymorphic site to the human genome and extract sequence information for 500 nucleotides on each side of the polymorphic sites. Next, they mapped human sequence to the chimpanzee genome to establish orthology. A parsimony method was then used to infer ancestral alleles and calculate the rate of CpG substitution.

Zhao and Jiang observed that the absolute value of the slope of the relationship between log (CpG substitution rate) and GC content *increased* with the length over which GC content was measured (segment lengths of 101 bps to 1001 bps around the CpG site, Figure 1C in Zhao and Jiang 2007). This result contradicts our results. However, there’s no explanation for this increase in slope in Zhao and Jiang (2007) hence it is hard to compare their conclusions to ours. Furthermore, their data and analyses methods differ from our approach. It is possible that the SNP database may suffer from ascertainment bias. Also, there are differences in analyses method, for example we removed GpCs that may overlap with CpGs (and vice versa). Another difference between the analysis of Zhao and Jiang (2007) and ours is their use of overlapping windows. They consider the effect of the G+C content in the flanking 101 nucleotides, and then contrast this with, for example, the effect of the G+C content in the flanking 501 nucleotides. However, the closest 101 nucleotides is being used in both calculations, which confounds its effect. In contrast, we use a sliding window analysis to reduce any confounding effects.

Their analyses may have been influenced by the drawback presented by the data or other bias. For example, Zhao and Jiang (2007)’s CpG and GpC rates are startlingly large. Specifically, in segments of 101 nucleotides, they report CpG rates over 0.7 whereas other studies report rates less than 0.2 (Nachman and Crowell 2000; Fryxell and Moon 2005; Meunier et al. 2005; Kim et al. 2006; Taylor et al. 2006), and they report GpC rates over 0.3, which is over an order of magnitude larger than other studies (Fryxell and Moon 2005).

**Supplementary Figure Legends**

**Figure S1: Sliding window analysis of the relationship between CpG substitution rate and normalized G+C content.** The same experiment as in Figure 3 with window size 25 and step size 5. **(A)** The distance decaying effect of G+C content on the rate of CpG substitution persists even with a smaller window size of 25 bps (as compared window size of 200 bps in Fig. 3). In the case of GpC sites, there was no distance decaying effect. (**B)**: Results of the chi-square test for the independence of the rate of CpG substitution and the G+C content of the windows. The blue line indicates log_10_ (*P*-value) = -1.30. The distance decaying effect subsided after ~2000 bps (**C)** Results of the same experiment as in panel B, but for GpC sites. There is no distance decaying effect.

**Figure S2: The distribution of GC content in 100kb segments around CpG and GpC sites. (A)** The G+C content of 100kb segments around CpG sites GC_global_ followed a bimodal distribution with means 39% and 48% respectively. The red line indicates GC_global =_ 43%, which was used as the cutoff to differentiate between low- GC_global_ and high-GC_global_  regions. **(B)** G+C content of 100kb segments around GpC sites also exhibited a bimodal distribution, with approximately the same means as those of GC_global_. The red line marks G+C content of 43%.

**Figure S3: Distance decaying relationship between G+C content and CpG substitution rate in low- GC_global_ regions.** Same analysis as in Figure 3 in the paper with CpG (and GpC) sites with G+C content of 100kb segments around them less than 43%. **(A)** The distance decaying effect of local G+C content on the rate of CpG substitutions was apparent, and the curves converged at ~1500 bps. In case of GpC, there was no distance decaying effect. **(B)** The test for independence of G+C content and the rate of CpG substitutions. The distance decaying effect was apparent from the gradual increase of *P*-values with increase in distance. P-Values become insignificant at ~ 1500 bps. **(C)** The results of the test for independence of G+C content and the rate of CpG substitutions. No distance decaying effect was observed between GpC substitution rate and G+C content.

**Figure S4: Relationship between G+C content and CpG substitution in high- GC_global_ regions.** Same analysis as in Supplementary Figure 2 with CpG (and GpC) sites with G+C content of 100kb segments around them greater than 43%. **(A)** The distance decaying effect for CpG sites was not apparent because of the fluctuations caused by reduced sample size in bins. In case of GpC, there was no distance decaying effect. **(B)** The test for dependence of CpG substitution rate and G+C content was insignificant starting at distances close to the CpG site. **(C)** No distance decaying effect was observed between GpC substitution rate and G+C content.

**Figure S5: Relationship between G+C content and substitution rate when CpG sites from introns were included.** Same analysis as in Figure 3 of the paper with CpG sites that lie within introns and that are at least 3kb away from exons included in the data set. The results are similar to that obtained in Figures 3 and 4 in the main text.

**References for Supplementary Text**

Benson DA, Karsch-Mizrachi I, Lipman DJ, Ostell J, Wheeler DL: GenBank. *Nucleic Acids Res* 2006, **34** (Database issue): D16-20.

Fryxell, K.J. and Moon, W.-J. 2005. CpG mutation rates in the human genome are highly dependent on local GC content. *Mol. Biol. Evol.* **22:** 650-658.

Fryxell, K.J. and Zuckerkandl, E. 2000. Cytosine deamination plays a primary role in the evolution of mammalian isochores. *Mol. Biol. Evol.* **17:** 1371-1383.

Kim, S.-H., Elango, N., Warden, C.W., Vigoda, E., and Yi, S. V. 2006. Heterogenous genomic molecular clocks in primates. *PLoS Genetics* **2:** e163.

Lander, E.S., Linton, L.M., Birren, B., Nusbaum, C., Zody, M.C., Baldwin, J., Devon, K., Dewar, K., Doyle, M., FitzHugh,W. et al. 2001. Initial sequencing and analysis of the human genome. *Nature* **409:** 860-921.

Meunier, J., Khelifi, A., Navratil, V., and Duret. L. 2005. Homology-dependent methylation in primate repetitive DNA. *Proc. Natl. Acad. Sci. USA.* **102:** 5471-5476.

Nachman, M.W., and Crowell, S.L. 2000. Estimate of the mutation rate per nucleotide in humans. *Genetics* **156:** 297-304.

Schwartz S, Kent WJ, Smit A, Zhang Z, Baertsch R, Hardison RC, Haussler D, Miller W: Human-mouse alignments with BLASTZ. *Genome Res.* 2003, **13:** 103-107.

Taylor, J., S. Tyekucheva, M. Zody, F. Chiaromonte, and K.D. Makova. 2006. Strong and weak male mutation bias at different sites in the primate genomes: insights from the human-chimpanzee comparison. *Mol. Biol. Evol.* **23:** 565-573.

Zhao, Z., and C. Jiang. 2007. Methylation-dependent transition rates are dependent on local sequence lengths and genomic regions. *Mol. Biol. Evol.* **24**:23-25
