# Supplementary figures and images for: Mutations of Different Molecular Origins Exhibit Contrasting Patterns of Regional Substitution Rate Variation
Source: PLoS Comput Biol. 2008 Feb 29;4(2):e1000015. doi: 10.1371/journal.pcbi.1000015 (PMC2265638; doi:10.1371/journal.pcbi.1000015)

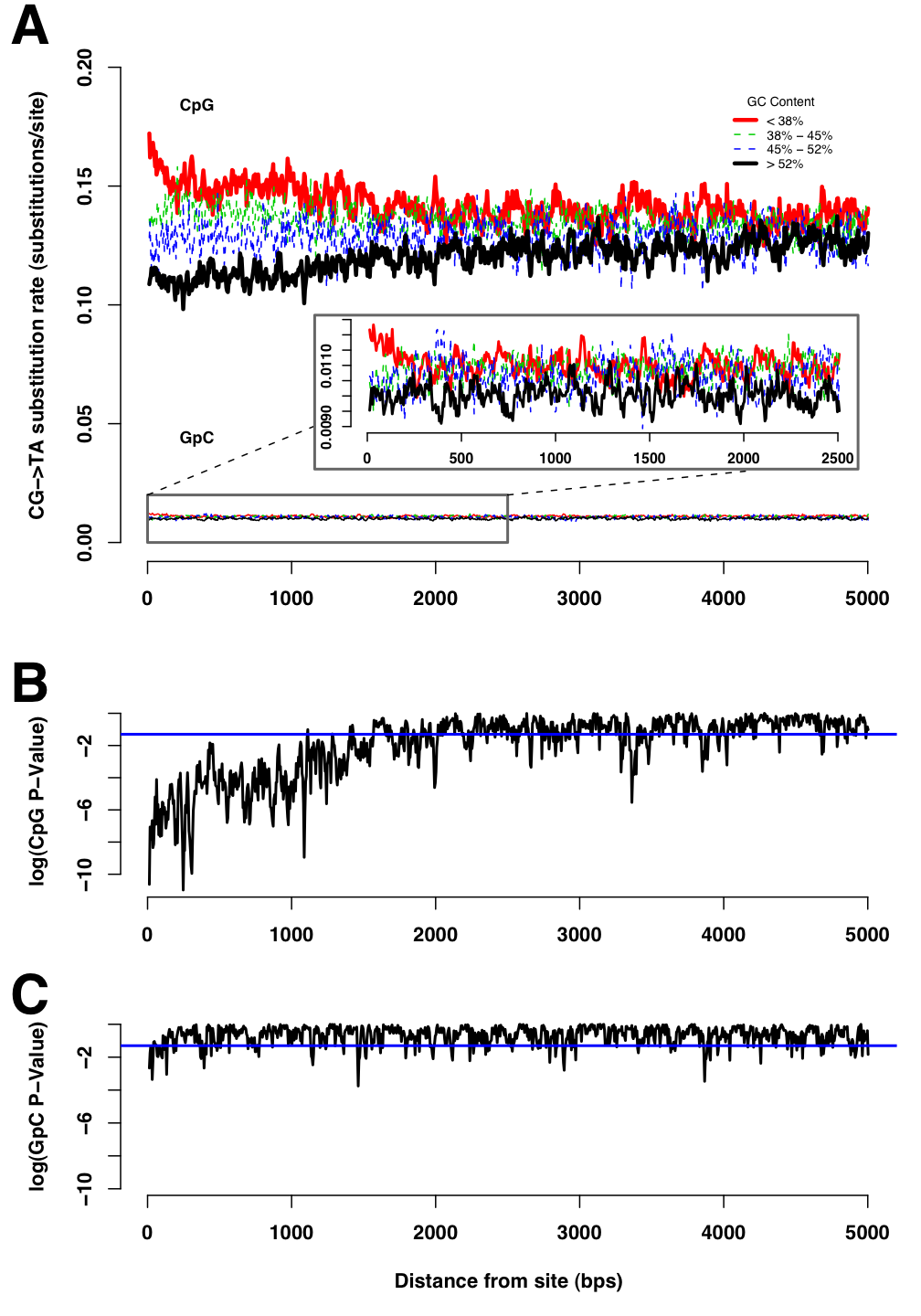

Supplement: Figure S1 — Sliding window analysis of the relationship between CpG substitution rate and normalized G+C content. The same experiment as in Figure 3 with window size 25 and step size 5. (A) The distance decaying effect of G+C content on the rate of CpG substitution persists even with a smaller window size of 25 bps (as compared window size of 200 bps in Figure 3). In the case of GpC sites, there was no distance decaying effect. (B) Results of the chi-square test for the independence of the rate of CpG substitution and the G+C content of the windows. The blue line indicates log10 (P-value) = −1.30. The distance decaying effect subsided after ∼2,000 bps. (C) Results of the same experiment as in (B), but for GpC sites. There is no distance-decaying effect. (4.36 MB TIF) [file pcbi.1000015.s002.tif]

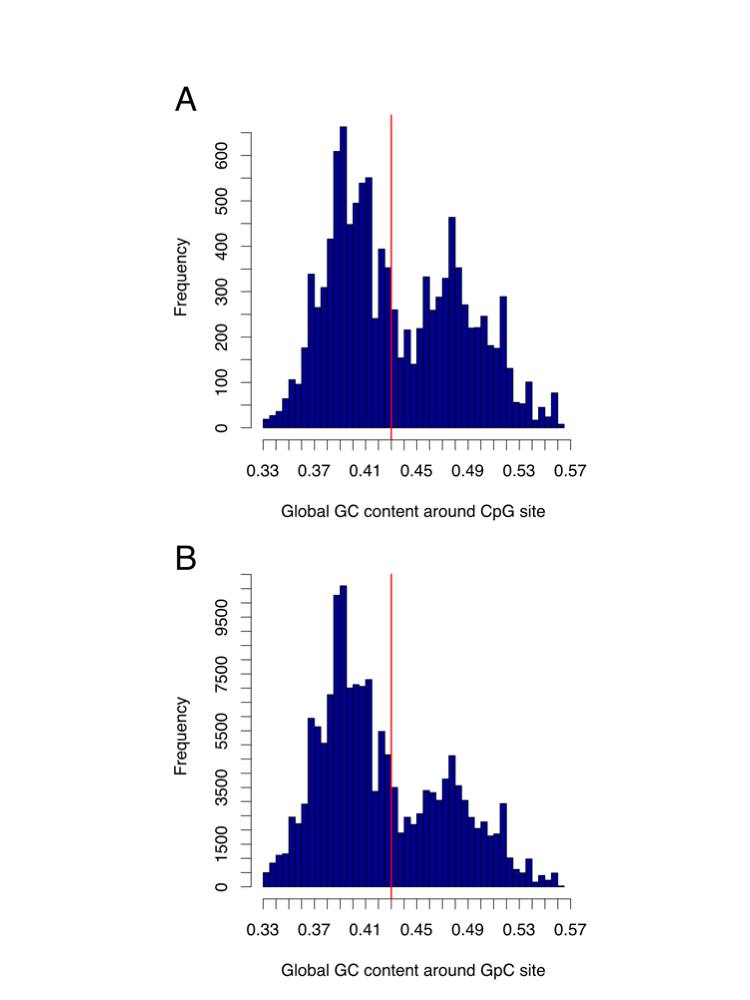

Supplement: Figure S2 — The distribution of GC content in 100 kb segments around CpG and GpC sites. (A) The G+C content of 100 kb segments around CpG sites GCglobal followed a bimodal distribution with means 39% and 48%, respectively. The red line indicates GCglobal = 43%, which was used as the cutoff to differentiate between low- GCglobal and high-GCglobal regions. (B) G+C content of 100 kb segments around GpC sites also exhibited a bimodal distribution, with approximately the same means as those of GCglobal. The red line marks G+C content of 43%. (0.29 MB TIF) [file pcbi.1000015.s003.tif]

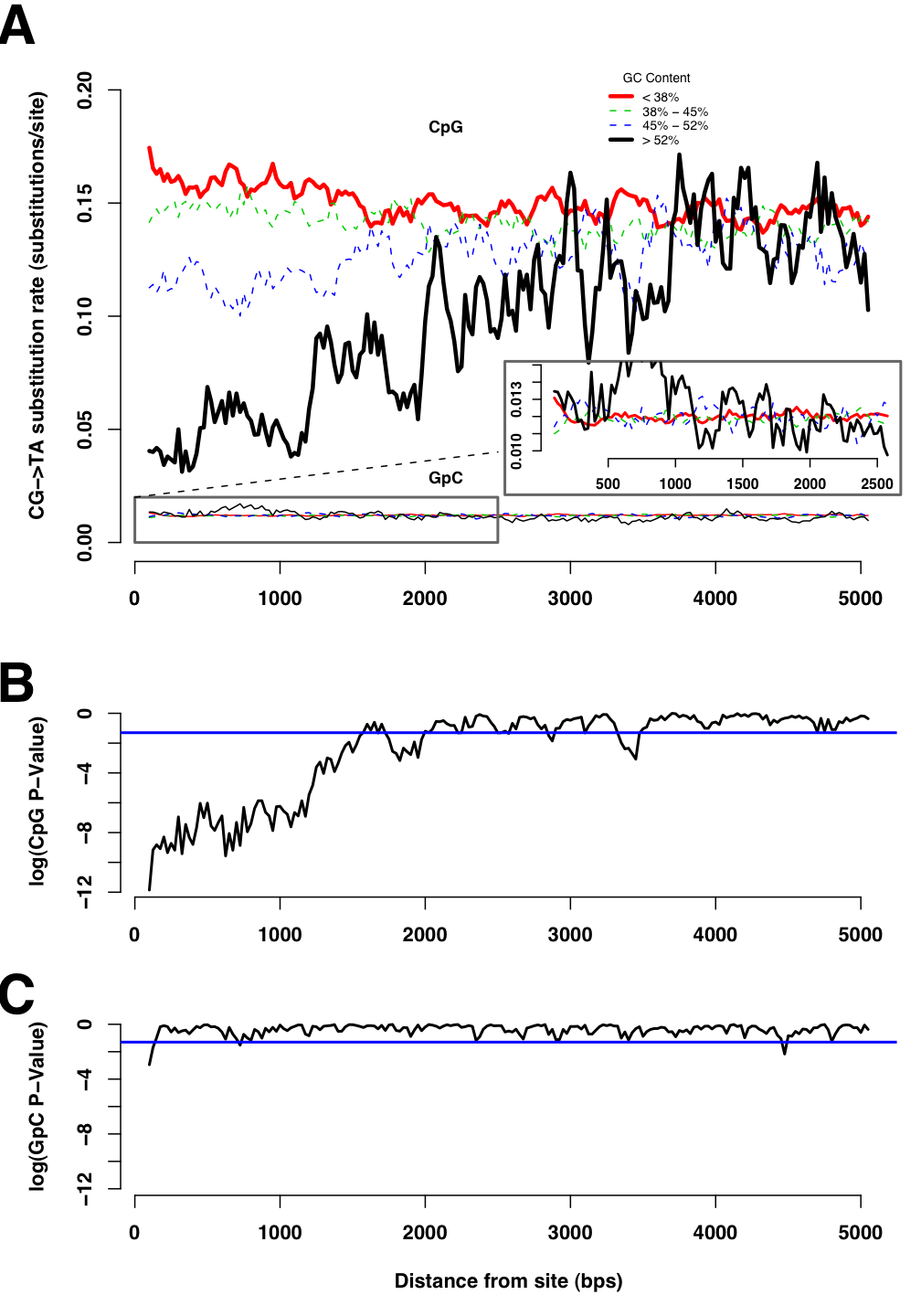

Supplement: Figure S3 — Distance decaying relationship between G+C content and CpG substitution rate in low- GCglobal regions. Same analysis as in Figure 3 in the paper with CpG (and GpC) sites with G+C content of 100 kb segments around them less than 43%. (A) The distance-decaying effect of local G+C content on the rate of CpG substitutions was apparent, and the curves converged at ∼1,500 bps. In case of GpC, there was no distance-decaying effect. (B) The test for independence of G+C content and the rate of CpG substitutions. The distance-decaying effect was apparent from the gradual increase of P-values with increase in distance. P-Values become insignificant at ∼1,500 bps. (C) The results of the test for independence of G+C content and the rate of CpG substitutions. No distance-decaying effect was observed between GpC substitution rate and G+C content. (4.36 MB TIF) [file pcbi.1000015.s004.tif]

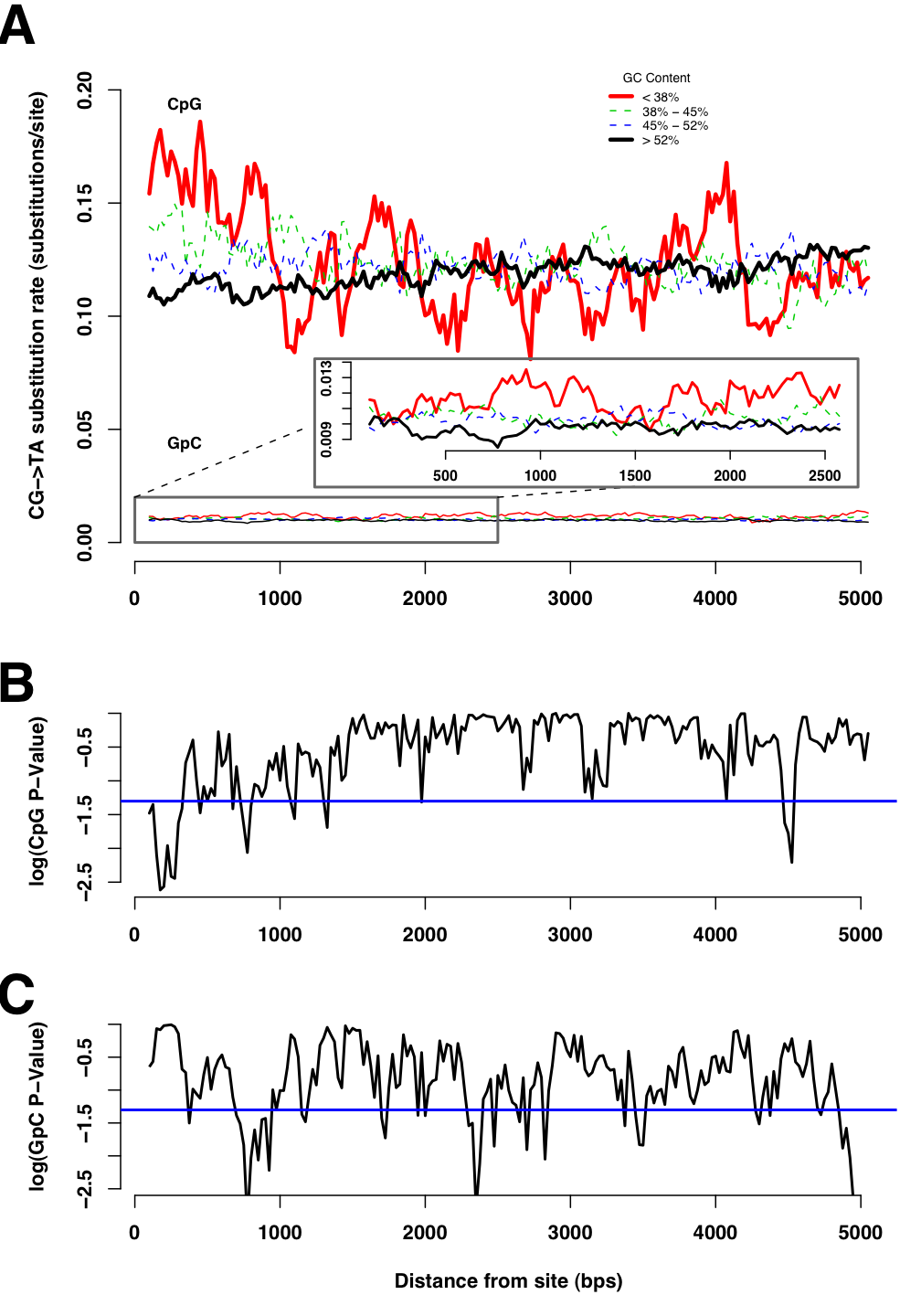

Supplement: Figure S4 — Relationship between G+C content and CpG substitution in high-GCglobal regions. Same analysis as in Figure S2 with CpG (and GpC) sites with G+C content of 100 kb segments around them greater than 43%. (A) The distance-decaying effect for CpG sites was not apparent because of the fluctuations caused by reduced sample size in bins. In case of GpC, there was no distance-decaying effect. (B) The test for dependence of CpG substitution rate and G+C content was insignificant starting at distances close to the CpG site. (C) No distance-decaying effect was observed between GpC substitution rate and G+C content. (4.36 MB TIF) [file pcbi.1000015.s005.tif]

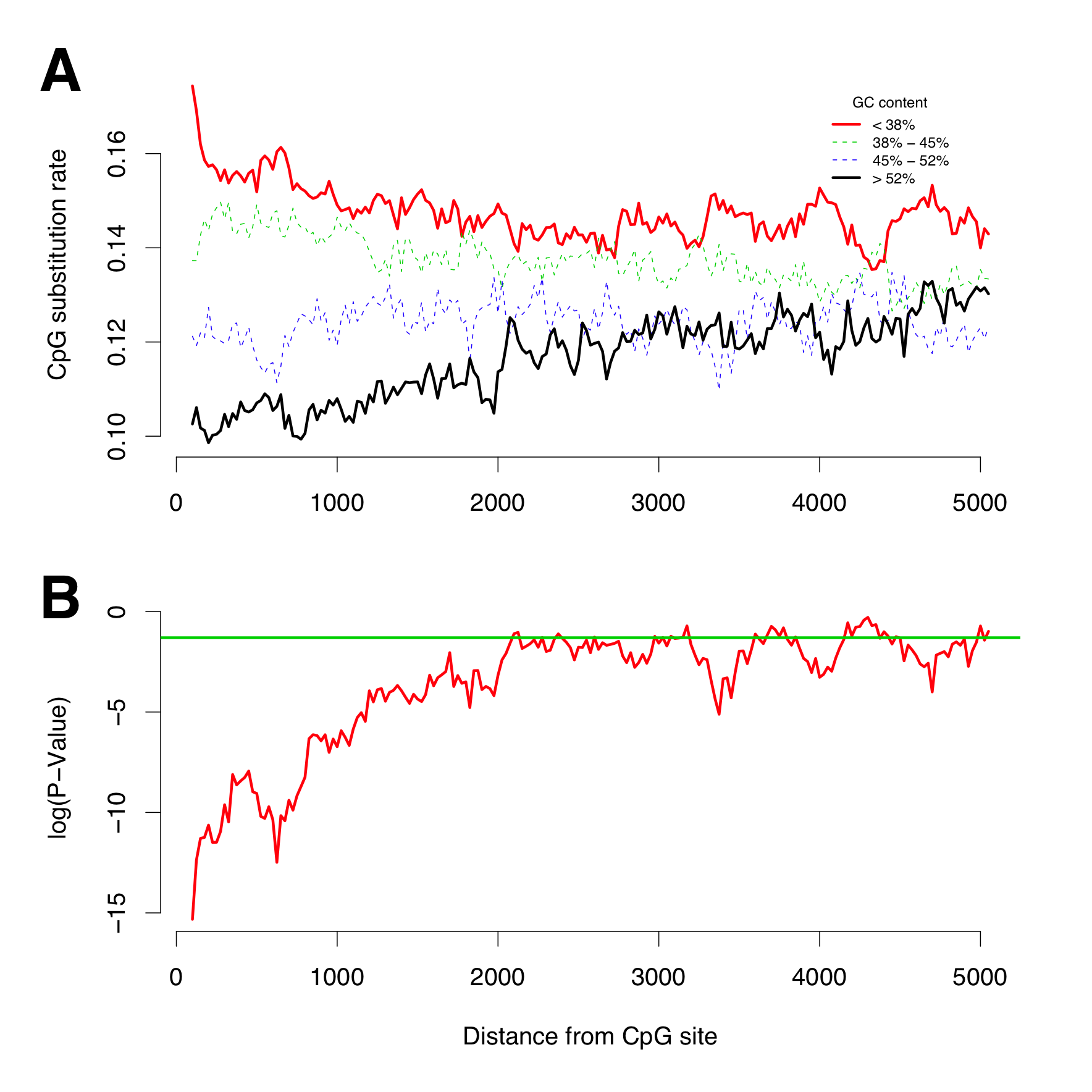

Supplement: Figure S5 — Relationship between G+C content and substitution rate when CpG sites from introns were included. Same analysis as in Figure 3 with CpG sites that lie within introns and that are at least 3 kb away from exons included in the data set. The results are similar to that obtained in Figures 3 and 4. (9.38 MB TIF) [file pcbi.1000015.s006.tif]
